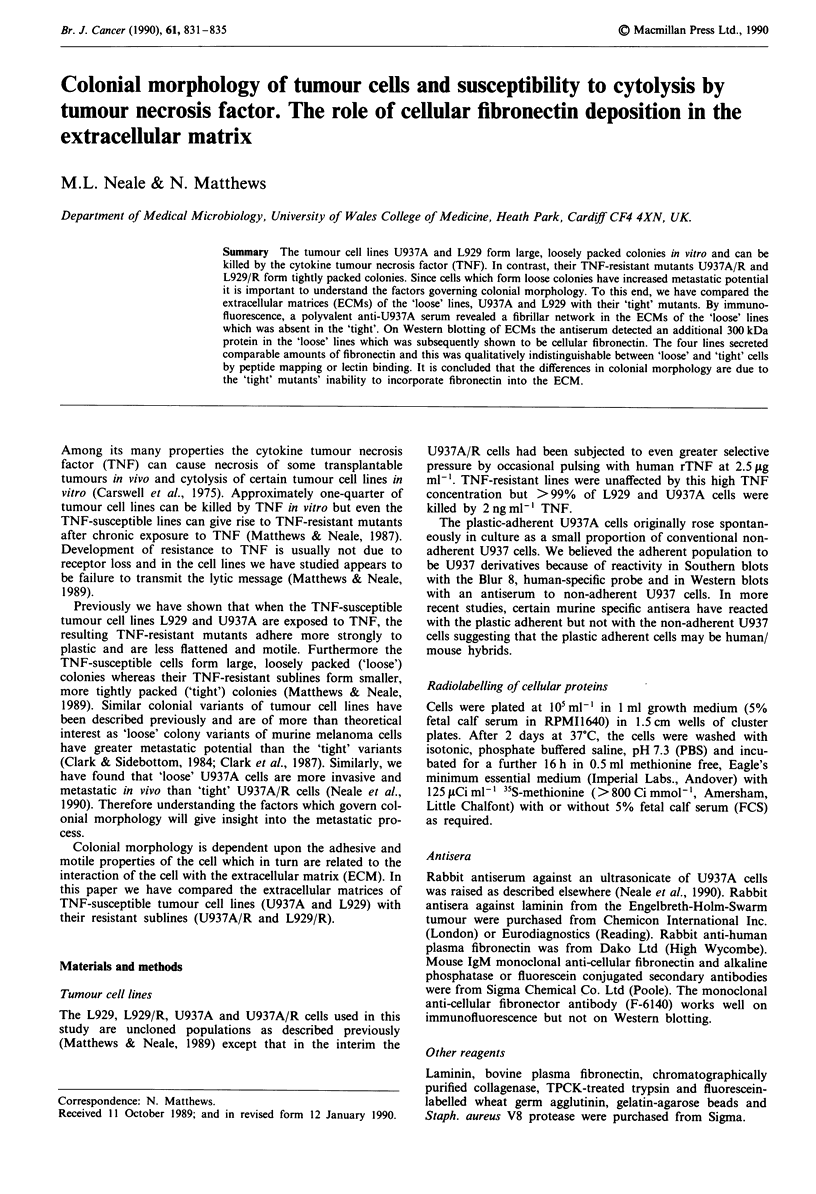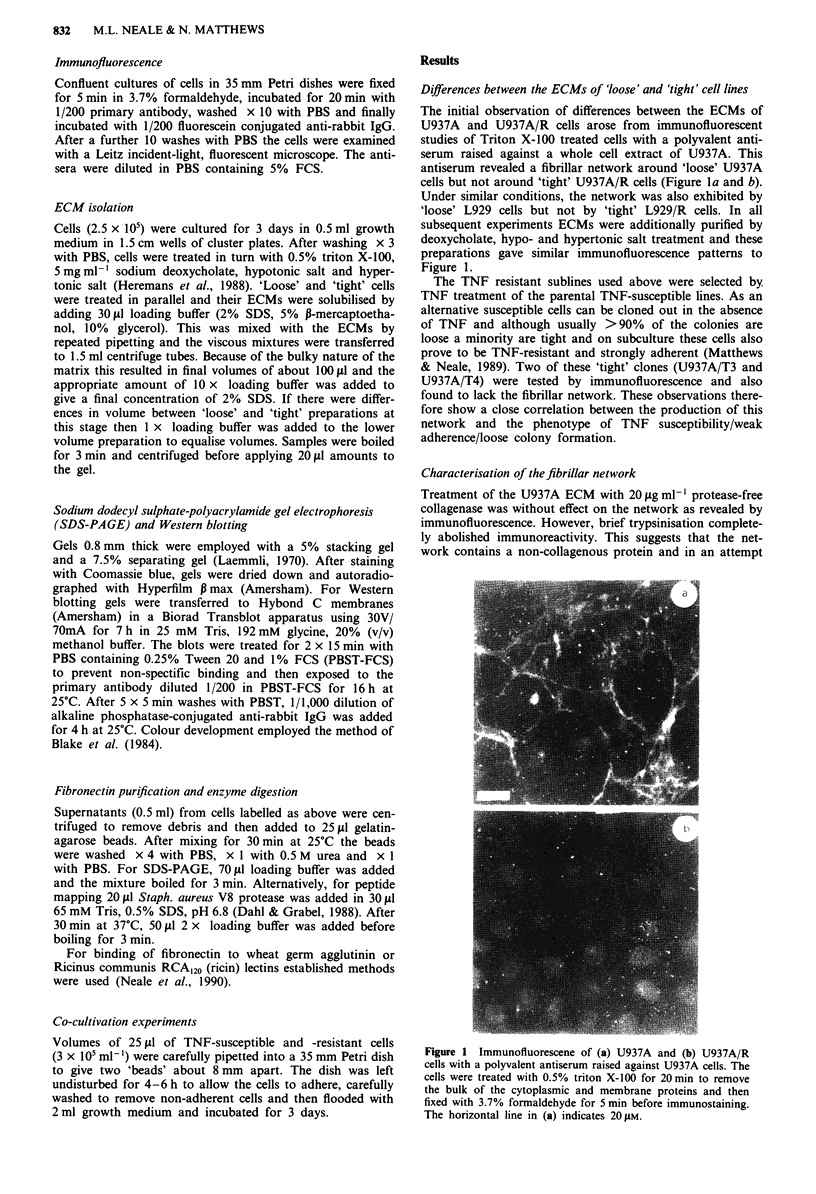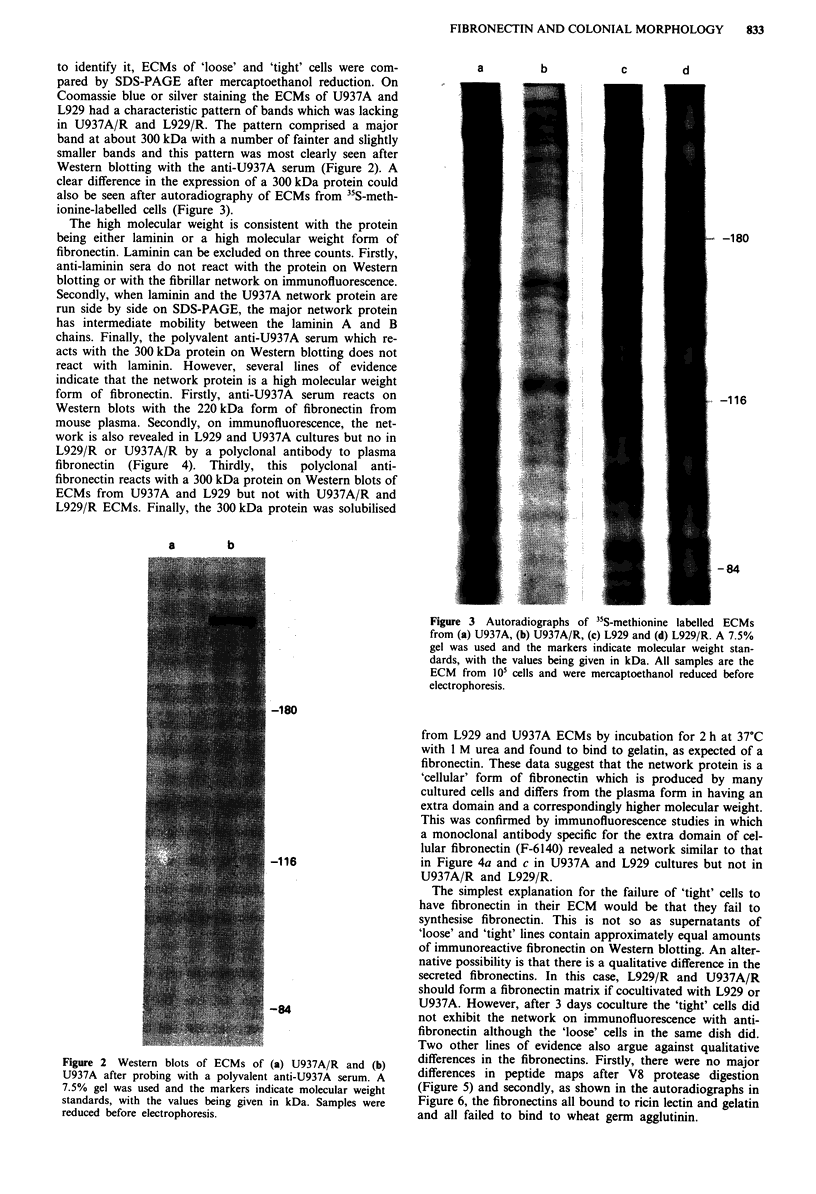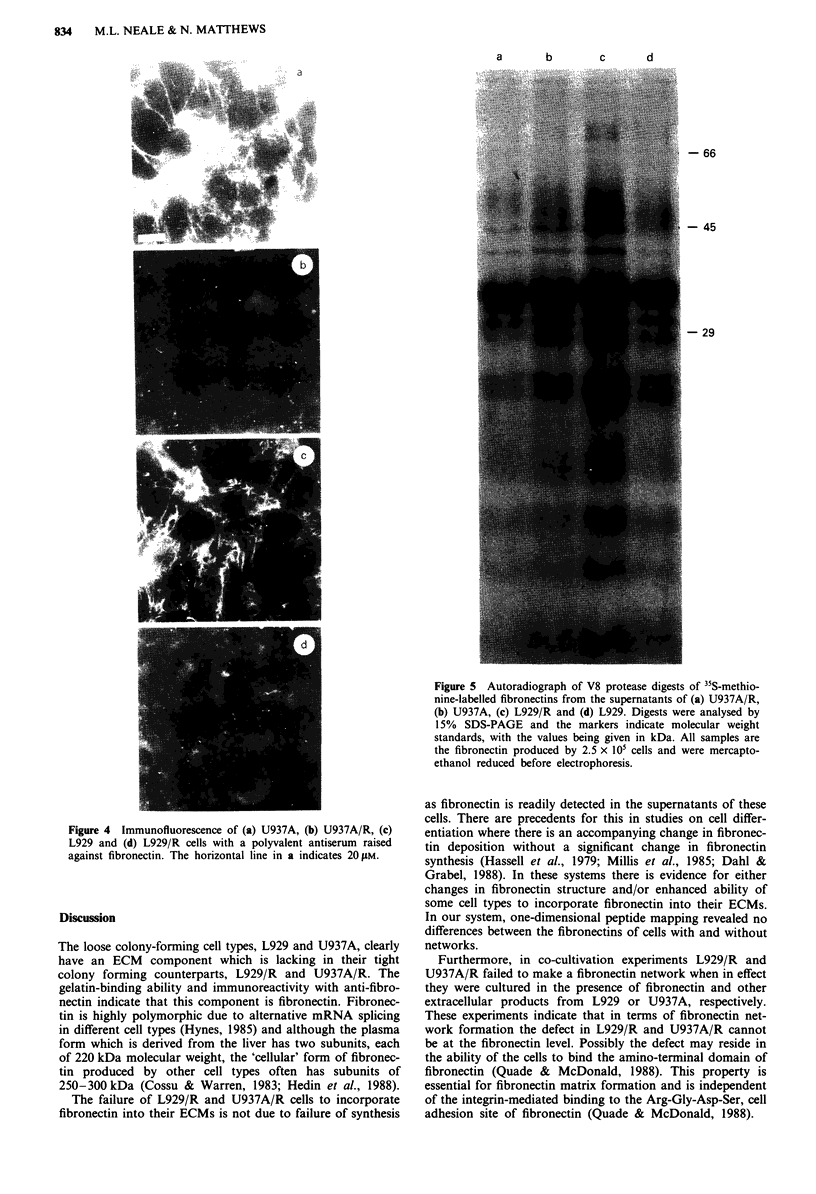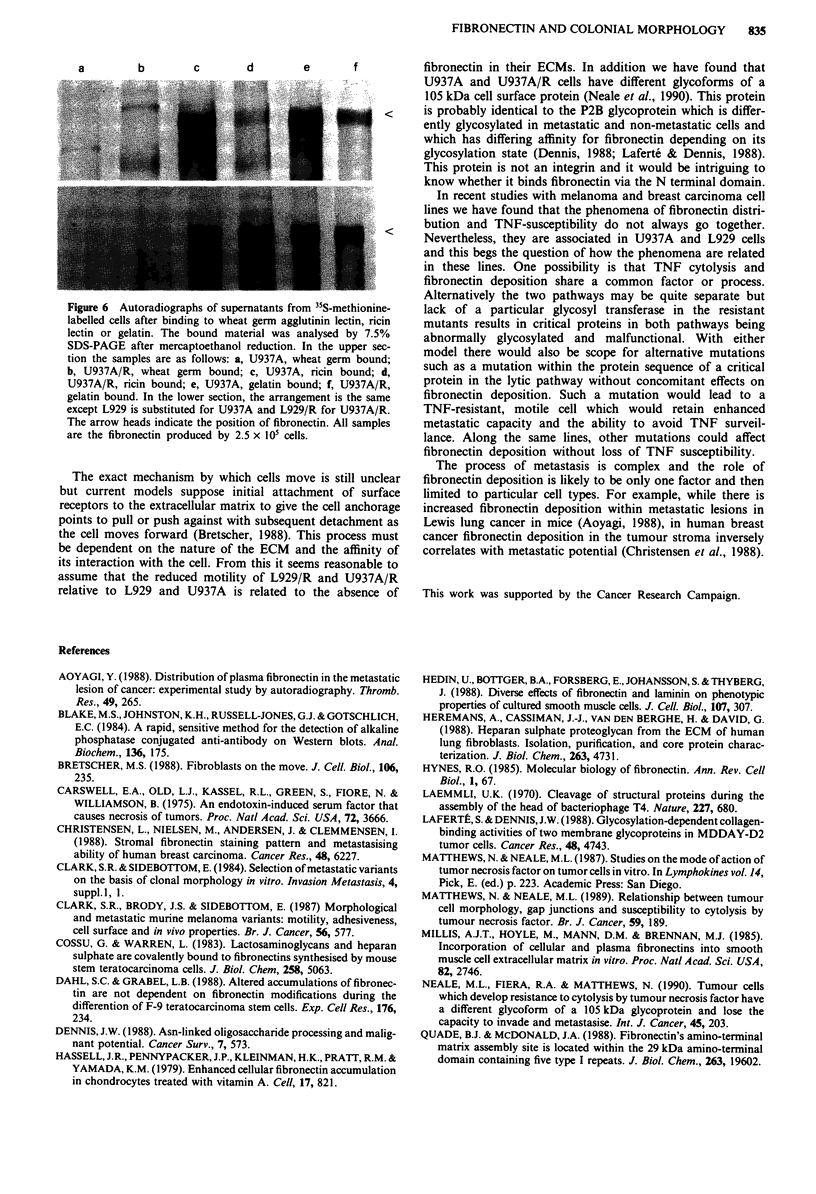# Colonial morphology of tumour cells and susceptibility to cytolysis by tumour necrosis factor. The role of cellular fibronectin deposition in the extracellular matrix.

**DOI:** 10.1038/bjc.1990.186

**Published:** 1990-06

**Authors:** M. L. Neale, N. Matthews

**Affiliations:** Department of Medical Microbiology, University of Wales College of Medicine, Heath Park, Cardiff, UK.

## Abstract

**Images:**


Br. J. Cancer (1990), 61, 831-835 © Macmillan Press Ltd., 1990

### Colonial morphology of tumour cells and susceptibility to cytolysis by tumour necrosis factor. The role of cellular fibronectin deposition in the extracellular matrix

M.L. Neale & N. Matthews

*Department of Medical Microbiology, University of Wales College of Medicine, Heath Park, Cardiff CF4 4XN, UK.*

**Summary** The tumour cell lines U937A and L929 form large, loosely packed colonies *in vitro* and can be killed by the cytokine tumour necrosis factor (TNF). In contrast, their TNF-resistant mutants U937A/R and L929/R form tightly packed colonies. Since cells which form loose colonies have increased metastatic potential it is important to understand the factors governing colonial morphology. To this end, we have compared the extracellular matrices (ECMs) of the 'loose' lines, U937A and L929 with their 'tight' mutants. By immunofluorescence, a polyvalent anti-U937A serum revealed a fibrillar network in the ECMs of the 'loose' lines which was absent in the 'tight'. On Western blotting of ECMs the antiserum detected an additional 300 kDa protein in the 'loose' lines which was subsequently shown to be cellular fibronectin. The four lines secreted comparable amounts of fibronectin and this was qualitatively indistinguishable between 'loose' and 'tight' cells by peptide mapping or lectin binding. It is concluded that the differences in colonial morphology are due to the 'tight' mutants' inability to incorporate fibronectin into the ECM.

Among its many properties the cytokine tumour necrosis factor (TNF) can cause necrosis of some transplantable tumours *in vivo* and cytolysis of certain tumour cell lines *in vitro* (Carswell *et al.*, 1975). Approximately one-quarter of tumour cell lines can be killed by TNF *in vitro* but even the TNF-susceptible lines can give rise to TNF-resistant mutants after chronic exposure to TNF (Matthews & Neale, 1987). Development of resistance to TNF is usually not due to receptor loss and in the cell lines we have studied appears to be failure to transmit the lytic message (Matthews & Neale, 1989).

Previously we have shown that when the TNF-susceptible tumour cell lines L929 and U937A are exposed to TNF, the resulting TNF-resistant mutants adhere more strongly to plastic and are less flattened and motile. Furthermore the TNF-susceptible cells form large, loosely packed ('loose') colonies whereas their TNF-resistant sublines form smaller, more tightly packed ('tight') colonies (Matthews & Neale, 1989). Similar colonial variants of tumour cell lines have been described previously and are of more than theoretical interest as 'loose' colony variants of murine melanoma cells have greater metastatic potential than the 'tight' variants (Clark & Sidebottom, 1984; Clark *et al.*, 1987). Similarly, we have found that 'loose' U937A cells are more invasive and metastatic *in vivo* than 'tight' U937A/R cells (Neale *et al.*, 1990). Therefore understanding the factors which govern colonial morphology will give insight into the metastatic process.

Colonial morphology is dependent upon the adhesive and motile properties of the cell which in turn are related to the interaction of the cell with the extracellular matrix (ECM). In this paper we have compared the extracellular matrices of TNF-susceptible tumour cell lines (U937A and L929) with their resistant sublines (U937A/R and L929/R).

### Materials and methods

### *Tumour cell lines*

The L929, L929/R, U937A and U937A/R cells used in this study are uncloned populations as described previously (Matthews & Neale, 1989) except that in the interim the U937A/R cells had been subjected to even greater selective pressure by occasional pulsing with human rTNF at 2.5 $\mu g\ ml^{-1}$. TNF-resistant lines were unaffected by this high TNF concentration but >99% of L929 and U937A cells were killed by 2 $ng\ ml^{-1}$ TNF.

The plastic-adherent U937A cells originally rose spontaneously in culture as a small proportion of conventional non-adherent U937 cells. We believed the adherent population to be U937 derivatives because of reactivity in Southern blots with the Blur 8, human-specific probe and in Western blots with an antiserum to non-adherent U937 cells. In more recent studies, certain murine specific antisera have reacted with the plastic adherent but not with the non-adherent U937 cells suggesting that the plastic adherent cells may be human/mouse hybrids.

### *Radiolabelling of cellular proteins*

Cells were plated at $10^5\ ml^{-1}$ in 1 ml growth medium (5% fetal calf serum in RPMI1640) in 1.5 cm wells of cluster plates. After 2 days at 37°C, the cells were washed with isotonic, phosphate buffered saline, pH 7.3 (PBS) and incubated for a further 16 h in 0.5 ml methionine free, Eagle's minimum essential medium (Imperial Labs., Andover) with 125 $\mu Ci\ ml^{-1}$ $^{35}S$-methionine (>800 Ci $mmol^{-1}$, Amersham, Little Chalfont) with or without 5% fetal calf serum (FCS) as required.

### *Antisera*

Rabbit antiserum against an ultrasonicate of U937A cells was raised as described elsewhere (Neale *et al.*, 1990). Rabbit antisera against laminin from the Engelbreth-Holm-Swarm tumour were purchased from Chemicon International Inc. (London) or Eurodiagnostics (Reading). Rabbit anti-human plasma fibronectin was from Dako Ltd (High Wycombe). Mouse IgM monoclonal anti-cellular fibronectin and alkaline phosphatase or fluorescein conjugated secondary antibodies were from Sigma Chemical Co. Ltd (Poole). The monoclonal anti-cellular fibronector antibody (F-6140) works well on immunofluorescence but not on Western blotting.

### *Other reagents*

Laminin, bovine plasma fibronectin, chromatographically purified collagenase, TPCK-treated trypsin and fluorescein-labelled wheat germ agglutinin, gelatin-agarose beads and *Staph. aureus* V8 protease were purchased from Sigma.

Correspondence: N. Matthews.
Received 11 October 1989; and in revised form 12 January 1990.

### *Immunofluorescence*

Confluent cultures of cells in 35 mm Petri dishes were fixed for 5 min in 3.7% formaldehyde, incubated for 20 min with 1/200 primary antibody, washed × 10 with PBS and finally incubated with 1/200 fluorescein conjugated anti-rabbit IgG. After a further 10 washes with PBS the cells were examined with a Leitz incident-light, fluorescent microscope. The antisera were diluted in PBS containing 5% FCS.

### *ECM isolation*

Cells ($2.5 \times 10^5$) were cultured for 3 days in 0.5 ml growth medium in 1.5 cm wells of cluster plates. After washing × 3 with PBS, cells were treated in turn with 0.5% triton X-100, 5 mg ml$^{-1}$ sodium deoxycholate, hypotonic salt and hypertonic salt (Heremans *et al.*, 1988). 'Loose' and 'tight' cells were treated in parallel and their ECMs were solubilised by adding 30 μl loading buffer (2% SDS, 5% β-mercaptoethanol, 10% glycerol). This was mixed with the ECMs by repeated pipetting and the viscous mixtures were transferred to 1.5 ml centrifuge tubes. Because of the bulky nature of the matrix this resulted in final volumes of about 100 μl and the appropriate amount of 10 × loading buffer was added to give a final concentration of 2% SDS. If there were differences in volume between 'loose' and 'tight' preparations at this stage then 1 × loading buffer was added to the lower volume preparation to equalise volumes. Samples were boiled for 3 min and centrifuged before applying 20 μl amounts to the gel.

### *Sodium dodecyl sulphate-polyacrylamide gel electrophoresis (SDS-PAGE) and Western blotting*

Gels 0.8 mm thick were employed with a 5% stacking gel and a 7.5% separating gel (Laemmli, 1970). After staining with Coomassie blue, gels were dried down and autoradiographed with Hyperfilm β max (Amersham). For Western blotting gels were transferred to Hybond C membranes (Amersham) in a Biorad Transblot apparatus using 30V/70mA for 7 h in 25 mM Tris, 192 mM glycine, 20% (v/v) methanol buffer. The blots were treated for 2 × 15 min with PBS containing 0.25% Tween 20 and 1% FCS (PBST-FCS) to prevent non-spectific binding and then exposed to the primary antibody diluted 1/200 in PBST-FCS for 16 h at 25°C. After 5 × 5 min washes with PBST, 1/1,000 dilution of alkaline phosphatase-conjugated anti-rabbit IgG was added for 4 h at 25°C. Colour development employed the method of Blake *et al.* (1984).

### *Fibronectin purification and enzyme digestion*

Supernatants (0.5 ml) from cells labelled as above were centrifuged to remove debris and then added to 25 μl gelatin-agarose beads. After mixing for 30 min at 25°C the beads were washed × 4 with PBS, × 1 with 0.5 M urea and × 1 with PBS. For SDS-PAGE, 70 μl loading buffer was added and the mixture boiled for 3 min. Alternatively, for peptide mapping 20 μl *Staph. aureus* V8 protease was added in 30 μl 65 mM Tris, 0.5% SDS, pH 6.8 (Dahl & Grabel, 1988). After 30 min at 37°C, 50 μl 2 × loading buffer was added before boiling for 3 min.

For binding of fibronectin to wheat germ agglutinin or Ricinus communis $RCA_{120}$ (ricin) lectins established methods were used (Neale *et al.*, 1990).

### *Co-cultivation experiments*

Volumes of 25 μl of TNF-susceptible and -resistant cells ($3 \times 10^5$ ml$^{-1}$) were carefully pipetted into a 35 mm Petri dish to give two 'beads' about 8 mm apart. The dish was left undisturbed for 4–6 h to allow the cells to adhere, carefully washed to remove non-adherent cells and then flooded with 2 ml growth medium and incubated for 3 days.

### Results

### *Differences between the ECMs of 'loose' and 'tight' cell lines*

The initial observation of differences between the ECMs of U937A and U937A/R cells arose from immunofluorescent studies of Triton X-100 treated cells with a polyvalent antiserum raised against a whole cell extract of U937A. This antiserum revealed a fibrillar network around 'loose' U937A cells but not around 'tight' U937A/R cells (Figure 1*a* and *b*). Under similar conditions, the network was also exhibited by 'loose' L929 cells but not by 'tight' L929/R cells. In all subsequent experiments ECMs were additionally purified by deoxycholate, hypo- and hypertonic salt treatment and these preparations gave similar immunofluorescence patterns to Figure 1.

The TNF resistant sublines used above were selected by TNF treatment of the parental TNF-susceptible lines. As an alternative susceptible cells can be cloned out in the absence of TNF and although usually >90% of the colonies are loose a minority are tight and on subculture these cells also prove to be TNF-resistant and strongly adherent (Matthews & Neale, 1989). Two of these 'tight' clones (U937A/T3 and U937A/T4) were tested by immunofluorescence and also found to lack the fibrillar network. These observations therefore show a close correlation between the production of this network and the phenotype of TNF susceptibility/weak adherence/loose colony formation.

### *Characterisation of the fibrillar network*

Treatment of the U937A ECM with 20 μg ml$^{-1}$ protease-free collagenase was without effect on the network as revealed by immunofluorescence. However, brief trypsinisation completely abolished immunoreactivity. This suggests that the network contains a non-collagenous protein and in an attempt

**Figure 1** Immunofluorescene of (**a**) U937A and (**b**) U937A/R cells with a polyvalent antiserum raised against U937A cells. The cells were treated with 0.5% triton X-100 for 20 min to remove the bulk of the cytoplasmic and membrane proteins and then fixed with 3.7% formaldehyde for 5 min before immunostaining. The horizontal line in (**a**) indicates 20 μM.

to identify it, ECMs of 'loose' and 'tight' cells were compared by SDS-PAGE after mercaptoethanol reduction. On Coomassie blue or silver staining the ECMs of U937A and L929 had a characteristic pattern of bands which was lacking in U937A/R and L929/R. The pattern comprised a major band at about 300 kDa with a number of fainter and slightly smaller bands and this pattern was most clearly seen after Western blotting with the anti-U937A serum (Figure 2). A clear difference in the expression of a 300 kDa protein could also be seen after autoradiography of ECMs from $^{35}S$-methionine-labelled cells (Figure 3).

The high molecular weight is consistent with the protein being either laminin or a high molecular weight form of fibronectin. Laminin can be excluded on three counts. Firstly, anti-laminin sera do not react with the protein on Western blotting or with the fibrillar network on immunofluorescence. Secondly, when laminin and the U937A network protein are run side by side on SDS-PAGE, the major network protein has intermediate mobility between the laminin A and B chains. Finally, the polyvalent anti-U937A serum which reacts with the 300 kDa protein on Western blotting does not react with laminin. However, several lines of evidence indicate that the network protein is a high molecular weight form of fibronectin. Firstly, anti-U937A serum reacts on Western blots with the 220 kDa form of fibronectin from mouse plasma. Secondly, on immunofluorescence, the network is also revealed in L929 and U937A cultures but no in L929/R or U937A/R by a polyclonal antibody to plasma fibronectin (Figure 4). Thirdly, this polyclonal anti-fibronectin reacts with a 300 kDa protein on Western blots of ECMs from U937A and L929 but not with U937A/R and L929/R ECMs. Finally, the 300 kDa protein was solubilised from L929 and U937A ECMs by incubation for 2 h at 37°C with 1 M urea and found to bind to gelatin, as expected of a fibronectin. These data suggest that the network protein is a 'cellular' form of fibronectin which is produced by many cultured cells and differs from the plasma form in having an extra domain and a correspondingly higher molecular weight. This was confirmed by immunofluorescence studies in which a monoclonal antibody specific for the extra domain of cellular fibronectin (F-6140) revealed a network similar to that in Figure 4*a* and *c* in U937A and L929 cultures but not in U937A/R and L929/R.

The simplest explanation for the failure of 'tight' cells to have fibronectin in their ECM would be that they fail to synthesise fibronectin. This is not so as supernatants of 'loose' and 'tight' lines contain approximately equal amounts of immunoreactive fibronectin on Western blotting. An alternative possibility is that there is a qualitative difference in the secreted fibronectins. In this case, L929/R and U937A/R should form a fibronectin matrix if cocultivated with L929 or U937A. However, after 3 days coculture the 'tight' cells did not exhibit the network on immunofluorescence with anti-fibronectin although the 'loose' cells in the same dish did. Two other lines of evidence also argue against qualitative differences in the fibronectins. Firstly, there were no major differences in peptide maps after V8 protease digestion (Figure 5) and secondly, as shown in the autoradiographs in Figure 6, the fibronectins all bound to ricin lectin and gelatin and all failed to bind to wheat germ agglutinin.

**Figure 2** Western blots of ECMs of (**a**) U937A/R and (**b**) U937A after probing with a polyvalent anti-U937A serum. A 7.5% gel was used and the markers indicate molecular weight standards, with the values being given in kDa. Samples were reduced before electrophoresis.

**Figure 3** Autoradiographs of $^{35}S$-methionine labelled ECMs from (**a**) U937A, (**b**) U937A/R, (**c**) L929 and (**d**) L929/R. A 7.5% gel was used and the markers indicate molecular weight standards, with the values being given in kDa. All samples are the ECM from $10^5$ cells and were mercaptoethanol reduced before electrophoresis.

**Figure 4** Immunofluorescence of (**a**) U937A, (**b**) U937A/R, (**c**) L929 and (**d**) L929/R cells with a polyvalent antiserum raised against fibronectin. The horizontal line in **a** indicates 20 μM.

**Figure 5** Autoradiograph of V8 protease digests of $^{35}$S-methionine-labelled fibronectins from the supernatants of (**a**) U937A/R, (**b**) U937A, (**c**) L929/R and (**d**) L929. Digests were analysed by 15% SDS-PAGE and the markers indicate molecular weight standards, with the values being given in kDa. All samples are the fibronectin produced by $2.5 \times 10^5$ cells and were mercaptoethanol reduced before electrophoresis.

### Discussion

The loose colony-forming cell types, L929 and U937A, clearly have an ECM component which is lacking in their tight colony forming counterparts, L929/R and U937A/R. The gelatin-binding ability and immunoreactivity with anti-fibronectin indicate that this component is fibronectin. Fibronectin is highly polymorphic due to alternative mRNA splicing in different cell types (Hynes, 1985) and although the plasma form which is derived from the liver has two subunits, each of 220 kDa molecular weight, the 'cellular' form of fibronectin produced by other cell types often has subunits of 250–300 kDa (Cossu & Warren, 1983; Hedin *et al.*, 1988).

The failure of L929/R and U937A/R cells to incorporate fibronectin into their ECMs is not due to failure of synthesis as fibronectin is readily detected in the supernatants of these cells. There are precedents for this in studies on cell differentiation where there is an accompanying change in fibronectin deposition without a significant change in fibronectin synthesis (Hassell *et al.*, 1979; Millis *et al.*, 1985; Dahl & Grabel, 1988). In these systems there is evidence for either changes in fibronectin structure and/or enhanced ability of some cell types to incorporate fibronectin into their ECMs. In our system, one-dimensional peptide mapping revealed no differences between the fibronectins of cells with and without networks.

Furthermore, in co-cultivation experiments L929/R and U937A/R failed to make a fibronectin network when in effect they were cultured in the presence of fibronectin and other extracellular products from L929 or U937A, respectively. These experiments indicate that in terms of fibronectin network formation the defect in L929/R and U937A/R cannot be at the fibronectin level. Possibly the defect may reside in the ability of the cells to bind the amino-terminal domain of fibronectin (Quade & McDonald, 1988). This property is essential for fibronectin matrix formation and is independent of the integrin-mediated binding to the Arg-Gly-Asp-Ser, cell adhesion site of fibronectin (Quade & McDonald, 1988).

**Figure 6** Autoradiographs of supernatants from $^{35}$S-methionine-labelled cells after binding to wheat germ agglutinin lectin, ricin lectin or gelatin. The bound material was analysed by 7.5% SDS-PAGE after mercaptoethanol reduction. In the upper section the samples are as follows: **a**, U937A, wheat germ bound; **b**, U937A/R, wheat germ bound; **c**, U937A, ricin bound; **d**, U937A/R, ricin bound; **e**, U937A, gelatin bound; **f**, U937A/R, gelatin bound. In the lower section, the arrangement is the same except L929 is substituted for U937A and L929/R for U937A/R. The arrow heads indicate the position of fibronectin. All samples are the fibronectin produced by $2.5 \times 10^5$ cells.

The exact mechanism by which cells move is still unclear but current models suppose initial attachment of surface receptors to the extracellular matrix to give the cell anchorage points to pull or push against with subsequent detachment as the cell moves forward (Bretscher, 1988). This process must be dependent on the nature of the ECM and the affinity of its interaction with the cell. From this it seems reasonable to assume that the reduced motility of L929/R and U937A/R relative to L929 and U937A is related to the absence of fibronectin in their ECMs. In addition we have found that U937A and U937A/R cells have different glycoforms of a 105 kDa cell surface protein (Neale *et al.*, 1990). This protein is probably identical to the P2B glycoprotein which is differently glycosylated in metastatic and non-metastatic cells and which has differing affinity for fibronectin depending on its glycosylation state (Dennis, 1988; Laferté & Dennis, 1988). This protein is not an integrin and it would be intriguing to know whether it binds fibronectin via the N terminal domain.

In recent studies with melanoma and breast carcinoma cell lines we have found that the phenomena of fibronectin distribution and TNF-susceptibility do not always go together. Nevertheless, they are associated in U937A and L929 cells and this begs the question of how the phenomena are related in these lines. One possibility is that TNF cytolysis and fibronectin deposition share a common factor or process. Alternatively the two pathways may be quite separate but lack of a particular glycosyl transferase in the resistant mutants results in critical proteins in both pathways being abnormally glycosylated and malfunctional. With either model there would also be scope for alternative mutations such as a mutation within the protein sequence of a critical protein in the lytic pathway without concomitant effects on fibronectin deposition. Such a mutation would lead to a TNF-resistant, motile cell which would retain enhanced metastatic capacity and the ability to avoid TNF surveillance. Along the same lines, other mutations could affect fibronectin deposition without loss of TNF susceptibility.

The process of metastasis is complex and the role of fibronectin deposition is likely to be only one factor and then limited to particular cell types. For example, while there is increased fibronectin deposition within metastatic lesions in Lewis lung cancer in mice (Aoyagi, 1988), in human breast cancer fibronectin deposition in the tumour stroma inversely correlates with metastatic potential (Christensen *et al.*, 1988).

This work was supported by the Cancer Research Campaign.

**References**

AOYAGI, Y. (1988). Distribution of plasma fibronectin in the metastatic lesion of cancer: experimental study by autoradiography. *Thromb. Res.*, **49**, 265.

BLAKE, M.S., JOHNSTON, K.H., RUSSELL-JONES, G.J. & GOTSCHLICH, E.C. (1984). A rapid, sensitive method for the detection of alkaline phosphatase conjugated anti-antibody on Western blots. *Anal. Biochem.*, **136**, 175.

BRETSCHER, M.S. (1988). Fibroblasts on the move. *J. Cell. Biol.*, **106**, 235.

CARSWELL, E.A., OLD, L.J., KASSEL, R.L., GREEN, S., FIORE, N. & WILLIAMSON, B. (1975). An endotoxin-induced serum factor that causes necrosis of tumors. *Proc. Natl Acad. Sci. USA*, **72**, 3666.

CHRISTENSEN, L., NIELSEN, M., ANDERSEN, J. & CLEMMENSEN, I. (1988). Stromal fibronectin staining pattern and metastasising ability of human breast carcinoma. *Cancer Res.*, **48**, 6227.

CLARK, S.R. & SIDEBOTTOM, E. (1984). Selection of metastatic variants on the basis of clonal morphology *in vitro*. *Invasion Metastasis*, **4**, suppl.1, 1.

CLARK, S.R., BRODY, J.S. & SIDEBOTTOM, E. (1987) Morphological and metastatic murine melanoma variants: motility, adhesiveness, cell surface and *in vivo* properties. *Br. J. Cancer*, **56**, 577.

COSSU, G. & WARREN, L. (1983). Lactosaminoglycans and heparan sulphate are covalently bound to fibronectins synthesised by mouse stem teratocarcinoma cells. *J. Biol. Chem*, **258**, 5063.

DAHL, S.C. & GRABEL, L.B. (1988). Altered accumulations of fibronectin are not dependent on fibronectin modifications during the differention of F-9 teratocarcinoma stem cells. *Exp. Cell Res.*, **176**, 234.

DENNIS, J.W. (1988). Asn-linked oligosaccharide processing and malignant potential. *Cancer Surv.*, **7**, 573.

HASSELL, J.R., PENNYPACKER, J.P., KLEINMAN, H.K., PRATT, R.M. & YAMADA, K.M. (1979). Enhanced cellular fibronectin accumulation in chondrocytes treated with vitamin A. *Cell*, **17**, 821.

HEDIN, U., BOTTGER, B.A., FORSBERG, E., JOHANSSON, S. & THYBERG, J. (1988). Diverse effects of fibronectin and laminin on phenotypic properties of cultured smooth muscle cells. *J. Cell. Biol.*, **107**, 307.

HEREMANS, A., CASSIMAN, J.-J., VAN DEN BERGHE, H. & DAVID, G. (1988). Heparan sulphate proteoglycan from the ECM of human lung fibroblasts. Isolation, purification, and core protein characterization. *J. Biol. Chem.*, **263**, 4731.

HYNES, R.O. (1985). Molecular biology of fibronectin. *Ann. Rev. Cell Biol.*, **1**, 67.

LAEMMLI, U.K. (1970). Cleavage of structural proteins during the assembly of the head of bacteriophage T4. *Nature*, **227**, 680.

LAFERTÉ, S. & DENNIS, J.W. (1988). Glycosylation-dependent collagen-binding activities of two membrane glycoproteins in MDDAY-D2 tumor cells. *Cancer Res.*, **48**, 4743.

MATTHEWS, N. & NEALE, M.L. (1987). Studies on the mode of action of tumor necrosis factor on tumor cells in vitro. In *Lymphokines vol. 14*, Pick, E. (ed.) p. 223. Academic Press: San Diego.

MATTHEWS, N. & NEALE, M.L. (1989). Relationship between tumour cell morphology, gap junctions and susceptibility to cytolysis by tumour necrosis factor. *Br. J. Cancer*, **59**, 189.

MILLIS, A.J.T., HOYLE, M., MANN, D.M. & BRENNAN, M.J. (1985). Incorporation of cellular and plasma fibronectins into smooth muscle cell extracellular matrix *in vitro*. *Proc. Natl Acad. Sci. USA*, **82**, 2746.

NEALE, M.L., FIERA, R.A. & MATTHEWS, N. (1990). Tumour cells which develop resistance to cytolysis by tumour necrosis factor have a different glycoform of a 105 kDa glycoprotein and lose the capacity to invade and metastasise. *Int. J. Cancer*, **45**, 203.

QUADE, B.J. & MCDONALD, J.A. (1988). Fibronectin's amino-terminal matrix assembly site is located within the 29 kDa amino-terminal domain containing five type I repeats. *J. Biol. Chem.*, **263**, 19602.